# Vitellogenin-2 Accumulation in the Fat Body and Hemolymph of *Babesia*-Infected *Haemaphysalis longicornis* Ticks

**DOI:** 10.3389/fcimb.2022.908142

**Published:** 2022-06-21

**Authors:** Maki Kuniyori, Nariko Sato, Naoaki Yokoyama, Shin-ichiro Kawazu, Xuenan Xuan, Hiroshi Suzuki, Kozo Fujisaki, Rika Umemiya-Shirafuji

**Affiliations:** ^1^ National Research Center for Protozoan Diseases, Obihiro University of Agriculture and Veterinary Medicine, Obihiro, Japan; ^2^ National Agricultural and Food Research Organization, Tsukuba, Japan

**Keywords:** tick, *Haemaphysalis longicornis*, vitellogenin, fat body, ovary, hemolymph, *Babesia*

## Abstract

The protozoan parasite *Babesia* spp. invades into tick oocytes and remains in the offspring. The transovarial transmission phenomenon of *Babesia* in ticks has been demonstrated experimentally, but the molecular mechanisms remain unclear. *Babesia* invasion into oocytes occurs along with the progression of oogenesis. In the present study, to find the key tick factor(s) for *Babesia* transmission, we focused on molecules involved in yolk protein precursor (vitellogenin, Vg) synthesis and Vg uptake, which are crucial events in tick oogenesis. With a *Haemaphysalis longicornis* tick–*Babesia ovata* experimental model, the expression profiles of *Akt*, *target of rapamycin*, *S6K*, *GATA*, and *Vg*, Vg synthesis-related genes, and Vg receptor (*VgR*) and autophagy-related gene 6 (*ATG6*), Vg uptake-related genes, were analyzed using real-time PCR using tissues collected during the preovipositional period in *Babesia*-infected ticks. The expression levels of *H. longicornis Vg-2* (*HlVg-2*) and *HlVg-3* decreased in the fat body of *Babesia*-infected ticks 1 day after engorgement. In the ovary, *HlVg-2* mRNA expression was significantly higher in *Babesia*-infected ticks than in uninfected ticks 1 and 2 days after engorgement and decreased 3 days after engorgement. *HlVgR* expression was significantly lower in *Babesia*-infected ticks than in uninfected ticks 2 and 4 days after engorgement. *HlATG6* had a lower gene expression in *Babesia*-infected ticks compared to uninfected ticks 2 days after engorgement. Additionally, western blot analysis using protein extracts from each collected tissue revealed that *H. longicornis* Vg-2 (HlVg-2) accumulate in the fat body and hemolymph of *Babesia*-infected ticks. These results suggest that Vg uptake from the hemolymph to the ovary was suppressed in the presence of *B. ovata*. Moreover, *HlVg-2* knockdown ticks had a lower detection rate of *B. ovata* DNA in the ovary and a significant reduction of *B. ovata* DNA in the hemolymph compared with control ticks. Taken together, our results suggest that accumulated HlVg-2 is associated with *Babesia* infection or transmission in the tick body. These findings, besides previous reports on VgR, provide important information to elucidate the transovarial transmission mechanisms of pathogens in tick vectors.

## 1 Introduction

*Haemaphysalis longicornis* ticks have a great ability to transmit various pathogens among vertebrates. Some pathogens, such as *Babesia* parasites, are transmitted transovarially in the tick, meaning that the parasites migrate from female ticks that fed on *Babesia*-infected animals to their offspring (Antunes et al., 2017). *Babesia* spp., intraerythrocytic protozoan parasites, infect mammals, including cattle, dogs, and humans (Chauvin et al., 2009; Schnittger et al., 2012). Babesiosis caused by *Babesia* spp. brings remarkable economic damage to the livestock industry worldwide. Blocking the life cycle of *Babesia*, which is established by tick feeding, is extremely important in controlling *Babesia* infectious diseases, especially bovine babesiosis. Female hard ticks lay thousands of eggs after taking a blood meal; thus, prevention of larval and female tick infestation is critical for the control of babesiosis. Although attenuated live vaccines against bovine babesiosis are available in some countries and the Bm86-based anti-tick vaccine is effective against *Rhipicephalus microplus* ticks (Schnittger et al., 2012; Rego et al., 2019; Ndawula and Tabor, 2020), other countries depend on chemical acaricides as a major prevention method. This leads to the increased emergence of acaricide-resistant ticks and health conditions caused by acaricide exposure in livestock and humans (De Vos and Bock, 2000; Domingos et al., 2013; Sivakumar et al., 2016; Vudriko et al., 2018). The development of novel therapies and vaccines against ticks and babesiosis remains important to be addressed globally.

The transovarial transmission of *Babesia* parasites in ticks has been demonstrated (Joyner et al., 1963; Donnelly and Peirce, 1975; Higuchi et al., 1991; Ohta et al., 1996). Several molecules involved in tick–*Babesia* interactions have been reported in many studies (reviewed by Antunes et al. (2017) and Mitchell et al. (2019)). It is believed that ovary molecules may be potential targets for vaccine development to block transovarial transmission of *Babesia*. Silencing the vitellogenin receptor (*VgR*) gene has been shown to lead to the absence of *Babesia* DNA in *H. longicornis* eggs (Boldbaatar et al., 2008) and in *Rhipicephalus microplus* larvae (Hussein et al., 2019). These findings suggest that *Babesia* parasites might invade the developing oocytes by interacting with tick VgR. Nevertheless, the molecular mechanisms underlying *Babesia* infection in tick ovaries remain unclear.

Vitellogenin (Vg), the yolk protein precursor, and its receptor, VgR, are key molecules in oogenesis and embryogenesis in ticks. Vg synthesis in the fat body is activated by target of rapamycin (TOR) signaling pathway upon engorgement in *H. longicornis* (Umemiya-Shirafuji et al., 2012a). Vg uptake from the hemolymph into oocytes *via* VgR is active at the preoviposition period after engorgement (Mihara et al., 2018; Umemiya-Shirafuji et al., 2019). Maeda et al. reported that *Babesia* parasites pass the midgut within 24 h after engorgement, migrate to the hemolymph and proliferate in other organs, including the ovary at the preoviposition period (Maeda et al., 2016). These findings lead us to hypothesize that there are interactions between oogenesis-related molecules, such as Vg and VgR, and *Babesia* molecules during the preovipositional period in *H. longicornis* ticks. On the other hand, Antunes et al. (2018) reported putative *Vg-3* in the salivary glands of *R. bursa* as a differentially expressed gene in response to blood feeding and *B. ovis* infection. Decreased blood uptake and increased *B. ovis* infection was observed in the salivary gland *Vg-3* knockdown ticks. However, the role of the salivary gland Vg-3 in *Babesia* transmission in the tick body is largely unknown.

In the present study, a tick–*Babesia* experimental infection model established previously (Maeda et al., 2016; Umemiya-Shirafuji et al., 2017) was used to explore the molecules involved in the transovarial transmission of the parasite in *H. longicornis*. The gene and protein expression of oogenesis-related molecules in *H. longicornis* (Boldbaatar et al., 2010a; Boldbaatar et al., 2010c; Umemiya-Shirafuji et al., 2012a; Umemiya-Shirafuji et al., 2012b) were examined after the semiartificial mouse skin membrane feeding of *Babesia*-infected bovine red blood cells. Based on the expression profiles, *Vg* gene silencing was conducted and the existence of *Babesia* DNA in the ovary and hemolymph was assessed.

## 2 Materials and methods

### 2.1 Ticks and Animals

The parthenogenetic *Haemaphysalis longicornis* (Okayama strain) was maintained by feeding on Japanese white rabbits (Japan SLC, Shizuoka, Japan) at the National Research Center for Protozoan Diseases, Obihiro University of Agriculture and Veterinary Medicine (OUAVM), Obihiro, Japan, as described previously (Umemiya-Shirafuji et al., 2019). Female ticks were used for all the experiments in the present study. BALB/c mice (specific-pathogen-free, 6 weeks old, female; CLEA Japan, Tokyo, Japan) were used for the semiartificial mouse skin membrane feeding. Rabbits and mice were maintained according to the guidelines approved by the OUAVM Animal Care and Use Committee (approval numbers: 18-11 and 18-12). The animals were reared in a temperature- and humidity-regulated room under controlled lighting, water, and commercial regular chow (CLEA Japan, Tokyo, Japan).

### 2.2 Parasites

Bovine blood was purchased from Japan Bio Serum (Tokyo, Japan) for the *in vitro* cultivation of the intraerythrocytic parasites, *Babesia ovata* (Miyake strain; Minami and Ishihara, 1980). After preparing red blood cells (RBCs), *B. ovata* was cultivated with RBCs in a sterilized mixture of GIT (Fujifilm Wako Pure Chemical, Osaka, Japan) and M199 media (Sigma-Aldrich, MO, USA) under a low-oxygen atmosphere, 5% O_2_, 5% CO_2_, and 90% N_2_, at 37°C as described previously (Igarashi et al., 1994; Maeda et al., 2016).

### 2.3 Collection of Organs From Engorged Female Ticks After Semiartificial Blood Feeding

Female *H. longicornis* were fed with *B. ovata*-infected RBCs (iRBCs) or *B. ovata*-free RBCs through a semiartificial feeding system as described previously (Umemiya-Shirafuji et al., 2017). First, unfed female ticks (five ticks per mouse) were allowed to feed on the back of a shaved mouse for 4–5 days. At the beginning of the rapid feeding period, the mouse skin around the blood sucking site was cut after euthanasia by the cervical dislocation and was used to make artificial feeding devices. Parasitemia was determined using Giemsa stained RBC smears before starting semiartificial feeding (Maeda et al., 2016). A mixture of 300 µL of *B. ovata*-iRBCs (parasitemia 1%–2%) or *B. ovata*-free RBCs and 700 µL of fetal bovine serum (BioWest, Nuaillé, France) was prewarmed at 30°C and was poured into each semiartificial feeding device. The RBC/serum mixture was replaced twice a day until engorgement. Engorged female ticks were collected and were kept in the dark in a glass container at 25°C until dissection. One to four days after engorgement (corresponds to the preovipositional period), the ticks were dissected under a stereomicroscope (SZX16; Olympus, Tokyo, Japan). By cutting the dorsal side of each tick slightly, hemolymph exuded from around the dorsal blood vessel under the cuticle was recovered using an individual tip sterilized. For RNA extraction, fat bodies and ovaries of 20 ticks in *Babesia*-iRBC and RBC groups, respectively, were collected individually into TRI reagent (Sigma-Aldrich, MO, USA). For protein extraction, fat bodies, ovaries, and hemocyte-containing hemolymphs were collected into the protein extraction solution explained below. The individual carcass was used for genomic DNA extraction to confirm the presence of *B. ovata* DNA as described in sections *Extraction of Genomic DNA and PCR and Nested PCR.*

### 2.4 Extraction of Genomic DNA

To detect the *B. ovata β-tubulin* gene, genomic DNA was extracted from the carcass using NucleoSpin^®^ Tissue Extraction kit (Takara Bio, Shiga, Japan), according to the manufacturer’s protocol, and from *B. ovata*-iRBCs using NucleoSpin^®^ Blood Extraction kit (Takara Bio, Shiga, Japan) as positive PCR control. All genomic DNA samples were treated with RNaseA (Life Technologies, CA, USA) before DNA extraction to avoid RNA contamination. The concentration of the purified DNA samples was measured using NanoDrop 2000 (Thermo Fisher Scientific, MA, USA). Samples were then stored at −30°C until use.

### 2.5 PCR and Nested PCR

Eighty nanograms of each carcass genomic DNA sample were used for the first amplification. A 20 µL reaction mixture contained 2 µL of 10× KOD-Plus-Neo PCR Buffer (Toyobo, Osaka, Japan), 2 µL of 2 mM dNTP, 1.2 µL of 25 mM MgSO_4_, 0.6 µL of 10 µM forward and reverse primers (*B.ovata*_tubulin_F2: 5′-GCATTGGGCACATGTCTAATCTG-3′; *B.ovata*_tubulin_R: 5′-CTCGCGGATCTTGCTGATCAGCAGA-3′ (Sivakumar et al., 2014)), 0.4 µL of KOD-Plus-Neo polymerase (1 U/µL) (Toyobo, Osaka, Japan) and a suitable amount of autoclaved distilled water. The PCR conditions were initial denaturation at 94°C for 2 min and 30 cycles of denaturation at 98°C for 10 s, annealing at 67.3°C for 30 s and extension at 68°C for 15 s. Next, 1 µL of each 50-fold-diluted first PCR amplicon was added to a 19 µL reaction mixture containing 2 µL of 10× KOD-Plus-Neo PCR Buffer, 2 µL of 2 mM dNTP, 1.2 µL of 25 mM MgSO_4_, 0.6 µL of 10 µM forward and reverse primers (*B.ovata*_tubulin_F: 5′-ACACTGTGCATCCTCACCGTCATAT-3′ (Sivakumar et al., 2014); *B.ovata*_tubulin_Rn2: 5′-CCTCAGCCTCCTTGCGTACAACATCAAG-3′ (Umemiya-Shirafuji et al., 2017)), 0.4 µL of KOD-Plus-Neo polymerase (1 U/µL) (Toyobo, Osaka, Japan) and 12.2 µL of autoclaved distilled water. The nested PCR conditions were 20 cycles of denaturation at 98°C for 10 s, annealing at 69.4°C for 30 s, and extension at 68°C for 15 s. Ribosomal DNA internal transcribed spacer region 2 was detected in *H. longicornis via* PCR, as described previously (Umemiya-Shirafuji et al., 2017). Each PCR product was subjected to agarose gel electrophoresis, was stained with ethidium bromide, and was detected under ultraviolet illumination. Subsequently, TA cloning was performed to confirm the amplicon sequences. The PCR product was incubated with a 10× A-attachment mix (Toyobo, Osaka, Japan) at 60°C for 10 min and was inserted into a pGEM-T easy vector (Promega, WI, USA). Each ligation solution was mixed with ECOS™ competent *Escherichia coli* DH5α (Nippon Gene, Tokyo, Japan) for transformation. The plasmid DNA was purified from positive transformants using NucleoSpin^®^ plasmid EasyPure (Takara Bio, Shiga, Japan), according to the manufacturer’s protocol. DNA sequencing was carried out by Fasmac (Kanagawa, Japan).

### 2.6 Real-time PCR

Using collected organs described in section *Collection of Organs From Engorged Female Ticks After Semiartificial Blood Feeding*, total RNA was extracted according to the Direct-zol™ RNA MiniPrep kit protocol (Zymo Research, CA, USA) and was treated with DNase (TURBO DNA-free™ Kit; Life Technologies, CA, USA) to remove genomic DNA. Five total RNA samples were pooled from the infected group and the noninfected group each day (one to four days after engorgement) and were concentrated using Ethachinmate (Nippon Gene, Tokyo, Japan). Single-stranded cDNA was synthesized using the total RNA and a ReverTra Ace^®^ qPCR RT Kit (Toyobo, Osaka, Japan). Real-time PCR was performed using THUNDERBIRD^®^ SYBR qPCR Mix and a 7300 Real-Time PCR System (Applied Biosystems, CA, USA), according to the manufacturer’s protocol. Target genes were *Akt*, *TOR*, *S6K*, *GATA*, *Vg-2*, and *Vg-3* in the fat body (Umemiya-Shirafuji et al., 2012a; Umemiya-Shirafuji et al., 2012b) and *VgR* and *ATG6* in the ovary (Kawano et al., 2011). Standard curves were created from five-fold serial dilutions of *H. longicornis* embryo cDNA. PCR was conducted with the following settings: 95°C for 10 min, 40 cycles of denaturation at 95°C for 15 s, and annealing/extension at 60°C for 60 s. The specificity of the PCR products was confirmed using dissociation curve analysis of the individual wells. The data were analyzed using the 7300 system SDS software version 1.4 (Applied Biosystems, CA, USA). In our study, the candidate internal control genes evaluated were *actin*, *L23* and *P0* of *H. longicornis*. After evaluating the expression stability of the candidate internal genes in all samples, *P0* (GenBank: EU048401) was the most stable one for real-time PCR. All reactions were run triplicate, and from the data, relative gene expression and the mean ± standard deviation (SD) were calculated using Microsoft Excel 2010.

### 2.7 Sodium Dodecyl Sulphate-Polyacrylamide Gel Electrophoresis and Western Blotting

The fat body, ovary, and hemocyte-containing hemolymph collected were suspended in Tris-buffered saline containing 1% Triton-X100 with cOmplete™, Mini, ethylenediaminetetraacetic acid (EDTA)-free protease inhibitor cocktail (Merck, Darmstadt, Germany), and phosphatase inhibitor (PhosSTOP™; Merck, Darmstadt, Germany). After gentle homogenization, the samples were incubated at 4°C for 24 h in a rotator. The extract was centrifuged at 21,640 ×g for 30 min at 4°C. The supernatant was transferred to a new tube and was stored at −30°C until use. Equal volumes of 2× sample buffer (4% sodium dodecyl sulfate, 20% glycerol, 125 mM Tris, 0.1% bromophenol blue, 10% 2-mercaptoethanol) were mixed with each protein extract. The mixtures were incubated at 95°C for 5 min. One microliter of the fat body- and ovary-derived protein extract per lane was separated under reducing conditions on a 7.5% gel (e-PAGEL; Atto, Tokyo, Japan). For the loading control, 5 μL of each protein extract per lane was used to detect *H. longicornis* β-tubulin protein (Umemiya-Shirafuji et al., 2012b). The protein concentration of the hemolymph-derived protein extract was measured according to the standard protocol of the Pierce™ BCA Protein Assay Kit (Thermo Fisher Scientific, MA, USA). To each lane, 5 ng of hemolymph sample was added and was separated on a gel under reducing conditions. Western blotting was performed using the antiserum against *H. longicornis* Vg-2 (Boldbaatar et al., 2010c). The signals were detected by incubation with alkaline phosphatase-labeled goat antimouse IgG H&L (Abcam, Cambridge, UK) and CDP-Star^®^ Detection Reagent (Cytiva, Tokyo, Japan). The images were captured with Image Quant LAS500 (Cytiva, Tokyo, Japan) and each signal was analyzed as integrated intensities of areas defined around the bands using Image J 1.51J8 software (National Institutes of Health, Bethesda, MD). The ratio between the band intensity of *H. longicornis* Vg-2 and β-tubulin was calculated using Microsoft Excel 2010.

### 2.8 Knockdown of *H. longicornis Vg-2* by RNA Interference

Using two sets of primers containing the T7 promoter sequence at the 5′ end (Vg-2 T7-Forward (5′- GGATCCTAATACGACTCACTATAGGCTTTGGAGAGTACTCCAAGAAC-3′ (Boldbaatar et al., 2010b)) and Vg-2-R2 (5′-ACGTAGCTGCTGGCGAA-3′); Vg-2-F (5′-CTTTGGAGAGTACTCCAAGAACTACAGGC-3′) and T7-Vg-2-R2 (5′-GGATCCTAATACGACTCACTATAGGACGTAGCTGCTGGCGAA-3′)), the 555-bp *Vg-2* gene fragment was amplified by PCR with KOD-Plus-Neo (Toyobo, Osaka, Japan) and *H. longicornis* fat body cDNA template derived from the first day after engorgement. The PCR products were purified using NucleoSpin^®^ Gel and PCR Clean-up (Takara Bio, Shiga, Japan) and were then used to generate double-stranded RNA (dsRNA) using the T7 RiboMax™ Express RNAi system (Promega, WI, USA), according to manufacturers’ protocols. Female ticks fixed on a glass slide with double-sided tape were injected with 0.5 μL of *H. longicornis Vg-2* dsRNA (1 μg/tick) or firefly *luciferase* (*Luc*) (Umemiya-Shirafuji et al., 2012a) dsRNA as control. The injected ticks (n = 29 for *Luc* dsRNA; n = 27 for *Vg-2* dsRNA) were placed in a 25°C incubator under continuous dark condition for 24 h and were then used for tick infestation on mice. As described above, semiartificial blood feeding was started at the rapid blood feeding period. All ticks of control and *Vg-2* RNAi groups were collected at engorgement and their body weight was measured. The individual ticks were dissected 2 days after engorgement to collect the fat body, ovary, and carcass. Hemolymph samples were also collected from the ticks to extract genomic DNA and proteins and were used for real-time PCR (see next section) and western blotting, respectively. Total RNA purified from the fat body was used to examine the *H. longicornis Vg-2* gene expression by real-time PCR as described in Section *Real-Time PCR*. Genomic DNA was extracted from the ovary using the NucleoSpin^®^ Tissue Extraction kit (Takara Bio, Shiga, Japan). The remaining individual carcass was immersed in 1 mL DNA extraction buffer (100 mM Tris-HCl (pH 8.0), 0.5% SDS, 100 mM NaCl, 10 mM EDTA (pH 8.0)). After homogenization, 20 µL of Proteinase K (20 mg/mL) (Fujifilm Wako Pure Chemical, Osaka, Japan) was added to each sample. These were incubated at 56°C overnight. Genomic DNA of the carcass homogenates were purified using Phenol/Chloroform/Isoamyl alcohol (25:24:1) (Nippon Gene, Tokyo, Japan) and Ethachinmate (Nippon Gene, Tokyo, Japan) and were dissolved in 5 mM Tris/HCl buffer (pH 8.5; Takara Bio, Shiga, Japan). To detect the *B. ovata β-tubulin* gene, 40 ng of ovary DNA and 50 ng of carcass DNA were subjected to PCR.

### 2.9 Detection of *Babesia ovata β-tubulin* Gene in the Hemolymph by Real-time PCR

To quantify the amount of *B. ovata β-tubulin*, real-time PCR was performed using KOD SYBR qPCR^®^ Mix (Toyobo, Osaka, Japan) and 40 ng of hemolymph genomic DNA as described in the section 2.8. To make the standard curve, PCR products of *B. ovata β-tubulin* obtained using a specific primer set (forward primer: 5′-ACACTGTGCATCCTCACCGTCATAT-3′; reverse primer: 5′-CTCGCGGATCTTGCTGATCAGCAGA-3′) described by Sivakumar et al. (2014) were cloned into pGEM-T easy vector (Promega, WI, USA). The plasmids were used as templates at 10-fold serial dilutions. PCR was performed under the following settings: 98°C for 2 min, 40 cycles of denaturation at 98°C for 10 s, annealing at 60°C for 10 s and extension at 68°C for 30 s. All reactions were run in triplicates. The relative abundance of *B. ovata* DNA and the mean ± standard deviation (SD) were calculated using Microsoft Excel 2010.

### 2.10 Statistical Analysis

Statistical analyses were performed using the two-factor factorial ANOVA and Tukey–Kramer method for gene expression analyses and Student’s t-test after confirming no interactions by two-factor factorial ANOVA for western blotting. To calculate the detection rate of *B. ovata* in the ovary and carcass, χ^2^ test was used. The differences in the body weight of engorged female ticks, knockdown efficiency, and the amount of *B. ovata β-tubulin* DNA in the hemolymph of the control and *H. longicornis Vg-2* RNAi groups were compared using Student’s t-test. The values obtained were expressed as mean ± SD. All calculations were conducted using Microsoft Excel 2010.

## 3 Results

### 3.1 Expression Profiles of Genes Involved in Vg Synthesis in the Fat Body and Ovary of *Babesia*-Infected Ticks

First, *B. ovata β-tubulin* was amplified by nested PCR using genomic DNA extracted from each carcass of the female ticks after semiartificial mouse skin membrane feeding with bovine RBCs (RBC group) or *Babesia*-infected bovine RBCs (*Babesia*-iRBC group). Samples in which amplification of the *B. ovata* gene was confirmed were used for subsequent experiments.

**Figure 1:**
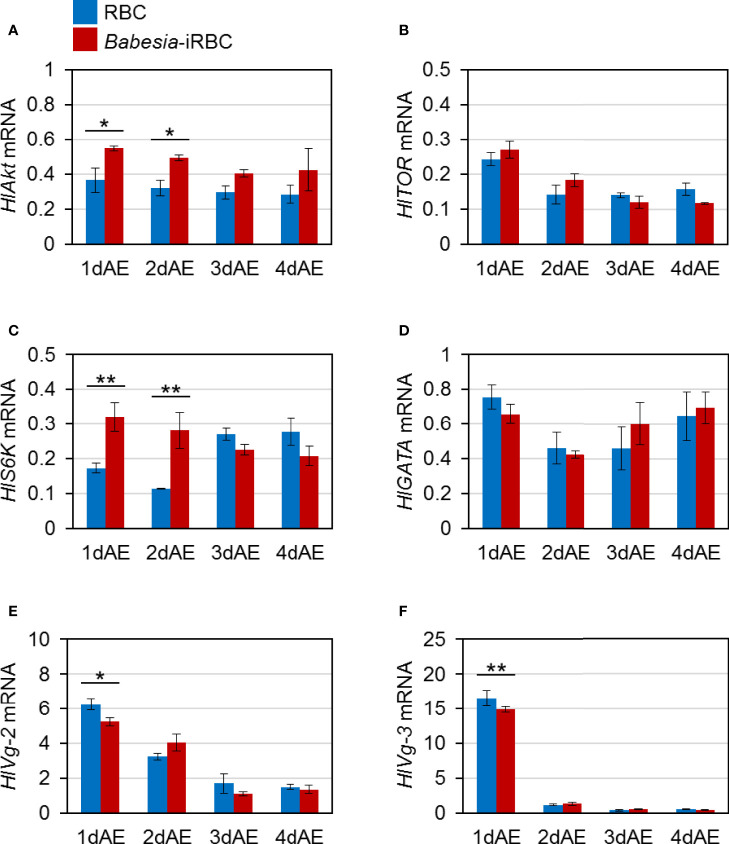
mRNA expression analysis of **(A)** *Akt* (*HlAkt*), **(B)** *target of rapamycin* (*HlTOR*), **(C)** *S6 kinase* (*HlS6K*), **(D)** *GATA* (*HlGATA*), **(E)** *vitellogenin-2* (*HlVg-2*), and **(F)** *vitellogenin-3* (*HlVg-3*) in the fat body after semiartificial mouse skin membrane feeding with bovine red blood cells (RBC group) or with *Babesia*-infected bovine red blood cells (*Babesia*-iRBC group). Total RNA was extracted from the fat body of engorged female ticks (n = 5) 1, 2, 3, and 4 days after engorgement (1dAE-4dAE). DNase-treated total RNA was used for cDNA synthesis. Data were normalized using *HlP0* levels in the cDNA samples. Data represent the mean ± SD of triplicated samples. Asterisks indicate the significant differences between the RBC and *Babesia*-iRBC groups (*P < 0.05, **P < 0.01 using the Tukey–Kramer method).

mRNA expression of *H. longicornis Akt* (*HlAkt*), *target of rapamycin* (*HlTOR*), *S6 kinase* (*HlS6K*), *GATA* (*HlGATA*), *vitellogenin-2* (*HlVg-2*), and *vitellogenin-3* (*HlVg-3*) in the fat body were examined *via* real-time PCR. These are *H. longicornis* genes involved in Vg synthesis that are expressed in the fat body. The expressions of *HlAkt* and *HlS6K* in the fat body were significantly upregulated in the *Babesia*-iRBC group 1 and 2 days after engorgement (**Figures 1A, C**) compared with the RBC group. The expression levels of *HlVg-2* and *HlVg-3* 1 day after engorgement decreased in the fat body of the *Babesia*-iRBC group compared with the RBC group (**Figures 1E, F**). There were no significant differences in the *HlTOR* and *HlGATA* expressions between the RBC and *Babesia*-iRBC groups (**Figures 1B, D**).

**Figure 2:**
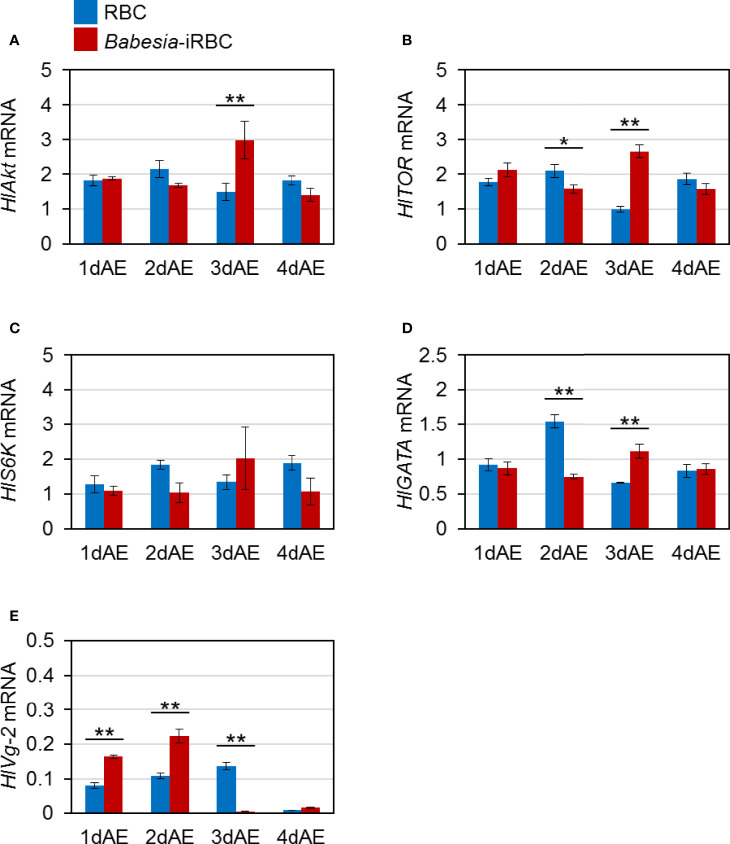
mRNA expression analysis of **(A)** *Akt* (*HlAkt*), **(B)** *target of rapamycin* (*HlTOR*), **(C)** *S6 kinase* (*HlS6K*), **(D)** *GATA* (*HlGATA*), and **(E)** *vitellogenin-2* (*HlVg-2*) in the ovary after semiartificial mouse skin membrane feeding with bovine red blood cells (RBC group) or with *Babesia*-infected bovine red blood cells (*Babesia*-iRBC group). Total RNA was extracted from the ovary of engorged female ticks (n = 5) 1, 2, 3, and 4 days after engorgement (1dAE-4dAE). The relative expression levels were determined by real-time PCR as shown in **Figure 1**. Data represent the mean ± SD of triplicated samples. Asterisks indicate the significant differences between the RBC and *Babesia*-iRBC groups (*P < 0.05, **P < 0.01 using the Tukey–Kramer method).

Next, mRNA expression of the following genes involved in Vg synthesis that is expressed in the ovary was analyzed *via* real-time PCR: *HlAkt*, *HlTOR*, *HlS6K*, *HlGATA*, and *HlVg-2*. *HlAkt* mRNA expression in the *Babesia*-iRBC group was upregulated 3 days after engorgement (**Figure 2A**). Decreased expressions of *HlTOR* and *HlGATA* were found in the *Babesia*-iRBC group 2 days after engorgement but increased in comparison with the RBC group 3 days after engorgement (**Figures 2B, D**). Interestingly, the expression level of *HlVg-2* was significantly higher in the *Babesia*-iRBC group than in the RBC group 1 and 2 days after engorgement but decreased 3 days after engorgement (**Figure 2E**). No significant difference in *HlS6K* expression was found between the *Babesia*-iRBC and RBC groups (**Figure 2C**).

### 3.2 Expression profiles of Genes Involved in Vg Uptake in the Ovary of *Babesia*-Infected Ticks

**Figure 3:**
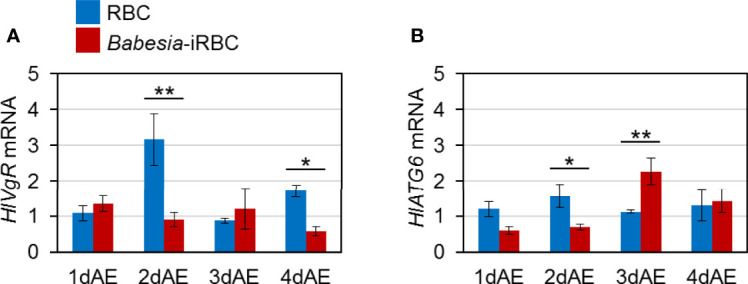
mRNA expression analysis of **(A)** vitellogenin receptor (*HlVgR*) and **(B)** autophagy-related gene 6 (*HlATG6*) in the ovary after semiartificial mouse skin membrane feeding with bovine red blood cells (RBC group) or with *Babesia*-infected bovine red blood cells (*Babesia*-iRBC group). Relative expression levels from extracted total RNA were determined by real-time PCR as shown in **Figure 2**. Data represent the mean ± SD of triplicated samples. Asterisks indicate the significant differences between the RBC and *Babesia*-iRBC groups (*P < 0.05, **P < 0.01 using the Tukey–Kramer method).

Expression levels of genes involved in Vg uptake, *HlVgR* and *HlATG6*, were analyzed using real-time PCR with the ovary cDNA. *HlVgR* expression level was significantly lower in the *Babesia*-iRBC group than in the RBC group 2 and 4 days after engorgement (**Figure 3A**). *HlATG6* had a lower gene expression in the *Babesia*-iRBC group than in the RBC group 2 days after engorgement but increased the next day (**Figure 3B**).

### 3.3 Detection of Vg-2 in *Babesia*-Infected Ticks

**Figure 4:**
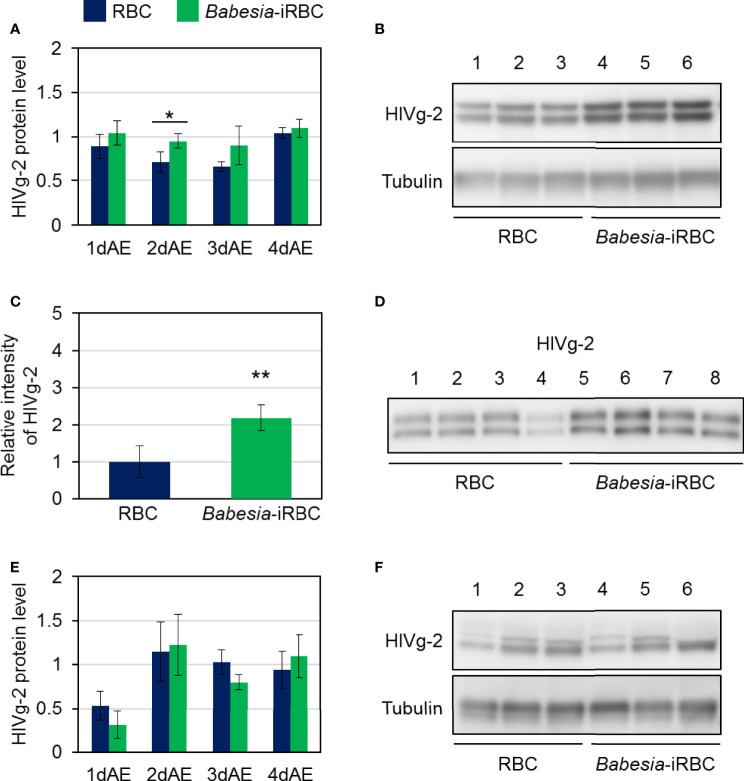
HlVg-2 protein levels in **(A, B)** the fat body, **(C, D)** the hemolymph, and **(E, F)** the ovary after semiartificial mouse skin membrane feeding with bovine red blood cells (RBC group) or with *Babesia*-infected bovine red blood cells (*Babesia*-iRBC group). The protein was extracted from the fat body or ovary of engorged female ticks 1, 2, 3, and 4 days after engorgement (1dAE-4dAE) and from the hemolymph 2dAE. Western blotting was performed using an anti-HlVg-2 serum. Anti-*H. longicornis* tubulin serum was used as loading control for the fat body and ovary samples. Positive bands of HlVg-2 in **(B)** the fat body and in **(F)** the ovary 2dAE are shown in the upper panels while bands of tubulin are in the lower panels. Lanes 1–6 contain equal numbers of tick fat body and ovary (n = 3 per group). HlVg-2 protein levels **(A, E)** were calculated relative to the band intensities of tubulin signals in the same samples. **(C, D)** HlVg-2 in the hemolymph 2dAE was detected using western blotting. Relative intensities **(C)** of HlVg-2 in the *Babesia*-iRBC and in the RBC group were calculated on the basis of the intensities of positive bands **(D)**. Lanes 1–8 contain equal numbers of tick hemolymph (n = 4 per group). Data represent the mean ± SD. Asterisks indicate the significant differences between the RBC and *Babesia*-iRBC groups (*P < 0.05, **P < 0.01 *via* Student’s t-test).

As there were significant differences in *HlVg-2* expression levels in both the fat body and ovary between the *Babesia*-iRBC and the RBC groups, the HlVg-2 protein levels in the fat body, hemolymph, and ovary were analyzed subsequently. Western blot analysis revealed that HlVg-2 protein levels in the fat body of the *Babesia*-iRBC group increased significantly 2 days after engorgement compared to the RBC group (**Figures 4A, B**). Using the hemolymph samples from 2 days after engorgement, HlVg-2 protein was also detected *via* western blotting. Since soluble components of the hemolymph were used as antigens for western blotting, relative intensities of HlVg-2 in the *Babesia*-iRBC group to the RBC group were calculated on the basis of the intensities of positive bands. The HlVg-2 protein level in the hemolymph of the *Babesia*-iRBC group was 2.2-fold higher than that of the RBC group 2 days after engorgement (**Figures 4C, D**). Conversely, in the ovary, HlVg-2 protein levels showed no significant difference between the *Babesia*-iRBC and RBC groups 1–4 days after engorgement (**Figures 4E, F**). These results suggest that HlVg-2 synthesized in the fat body and HlVg-2 secreted into the hemolymph accumulated in both tissues of *Babesia*-infected ticks. Combining with data from the western blot and **Figure 3**, it appears that *Babesia* infection might lead to attenuation of Vg uptake from the hemolymph to the ovary.

### 3.4 Detection of *B. ovata* in *Vg-2* Gene Knockdown Ticks

**Figure 5:**
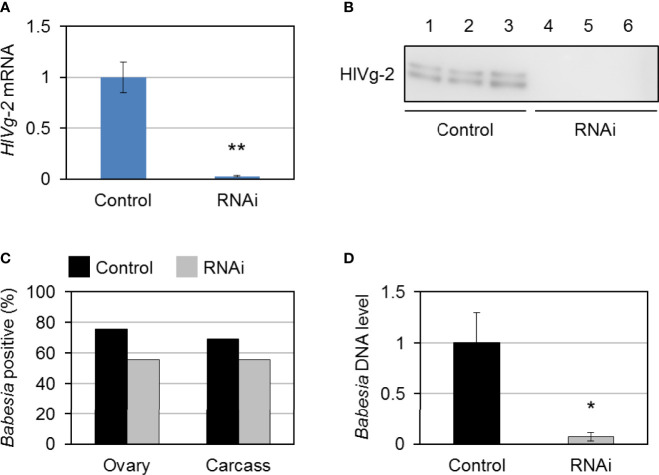
*HlVg-2* knockdown by RNA interference (RNAi) in *Babesia*-infected bovine red blood cells-fed *H longicornis* female ticks. **(A)** Total RNA was purified from the fat body 2 days after engorgement (control group, n = 29; *HlVg-2* RNAi group: n = 30). Knockdown efficacy was assessed *via* real-time PCR. Data represent the mean ± SD. Asterisks indicate the significant differences between the control and RNAi groups (**P < 0.01 *via* Student’s t-test). **(B)** HlVg-2 protein detection in the hemolymph of the control and the *HlVg-2* RNAi group *via* western blotting using an antibody against HlVg-2. Lanes 1–6 contain equal numbers of ticks (n = 3 per group). **(C)** *B. ovata* detection rate in the ovaries (control, n = 29; *HlVg-2* RNAi, n = 27) and carcass (control, n = 29; *HlVg-2* RNAi, n = 27). **(D)** Relative abundance of *B. ovata* DNA in the hemolymph (control, n = 4; *HlVg-2* RNAi, n = 7). Data represent the mean ± SD. Asterisks indicate the significant differences between the control and RNAi groups (*P < 0.05 *via* Welch's t-test).

To investigate the role of HlVg-2 on *B. ovata* infection and transmission in the tick body, *HlVg-2* gene silencing mediated by RNAi was performed. *HlVg-2* gene expression in the fat body of *HlVg-2* dsRNA-injected female ticks (*HlVg-2* RNAi group) was suppressed by 95.1% ± 2.3% compared with the dsRNA of *Luc*-injected female ticks (control group) (**Figure 5A**). Additionally, positive bands of HlVg-2 were detected in the western blot using anti-HlVg-2 antiserum in the hemolymph of the control group but not from the *HlVg-2* RNAi group (**Figure 5B**). These data suggest that injection of *HlVg-2* dsRNA induced silencing of *HlVg-2* effectively, resulting in a reduced amount of HlVg-2 protein in the hemolymph 2 days after engorgement. The mean body weight of engorged female ticks after semiartificial mouse skin membrane feeding with *Babesia*-iRBC was 117.0 ± 40.4 mg in the *HlVg-2* RNAi group and 128.0 ± 41.9 mg in the control group, with no significant difference between the two. *B. ovata β-tubulin* was amplified by nested PCR using the ovary and carcass samples of both groups 2 days after engorgement. In the ovary, the detection rate of *B. ovata β-tubulin* was 75.9% (22/29) in the control group while it was 55.6% (15/27) in the *HlVg-2* RNAi group (*P* = 0.07) (**Figure 5C**). In the carcass, the positive rate of *B. ovata* DNA was 69.0% (20/29) in the control group and 55.6% (15/27) in the *HlVg-2* RNAi group (*P* = 0.45). Although there are no significant differences in the *Babesia* DNA detection rates between the control and *HlVg-2* RNAi groups, it appeared that *HlVg-2* knockdown might cause a lower *Babesia* DNA detection rate. Hemolymph DNA samples from each group 2 days after engorgement were subjected to real-time PCR to assess the abundance of *B. ovata* DNA. Interestingly, *B. ovata* DNA in the hemolymph of the *HlVg-2* RNAi group was significantly lower than that of the control (**Figure 5D**). These data suggest that the abundance of *Babesia* in the hemolymph depends on the presence of HlVg-2 in *H. longicornis*.

## 4 Discussion

Having a transovarial transmission system, arthropod vectors play an important role in the persistence of the life cycle of microorganisms, including viruses, bacteria, and protozoan parasites. The transovarial transmission mechanism of pathogens has been elucidated in several species of vector arthropods. It has been reported that the rice stripe virus, a plant virus, binds to the insect vector’s Vg produced by the fat body to form a complex and invades the egg tubes of the ovariole (Huo et al., 2014). A subsequent study revealed that Vg produced by and processed in insect hemocytes interact with rice stripe virus (Huo et al., 2018). Specific interactions between the coat protein of tomato yellow leaf curl virus and whitefly Vg has been found to be vital for viral entry into the ovary of the whitefly *Bemisia tabaci* (Wei et al., 2017). Aside from these plant virus–insect vector relationships, it has also been reported that the honey bee *Apis mellifera* Vg binds to both Gram-positive and Gram-negative bacteria with pathogen-associated molecular patterns (Salmela et al., 2015). This study also demonstrated that Vg is required for the transport of cell wall pieces of bacteria into honeybee eggs. In blood-sucking arthropods, Ahmed et al. (2001) reported on the *Anopheles gambiae* mosquito *Vg* expression during *Plasmodium yoelii nigeriensis* infection. Twenty-four hours after blood feeding, *Plasmodium*-infected *Anopheles* mosquitoes had lower Vg synthesis, lower amounts of Vg in the hemolymph and vitellin in the ovary, and reduced egg production than uninfected mosquitoes. Our data were inconsistent with this mosquito study. This might be due to the phenomenon that *Plasmodium* parasites are not transmitted transovarially in mosquitoes, unlike *Babesia* in ticks. *Plasmodium* parasites take up the lipid transport protein lipophorin (Lp), a type of lipoprotein, for oocyst formation and *Vg* gene expression (Atella et al., 2009; Rono et al., 2010). The amount of Vg synthesized likely decreased in *Plasmodium*-infected mosquitoes because *Plasmodium* parasites utilize the Lp of mosquitoes. Little is known about the existence of Lp in ticks and the involvement of tick Vg in the transmission of tick-borne pathogens is also largely unknown. In the present study, we hypothesized that Vg may be associated with the transmission of *Babesia* parasites in the tick body and found that *HlVg-2*/HlVg-2 levels were elevated in *B. ovata*-infected *H. longicornis*.

Almost all *Babesia* species can infect the tick ovary and the phenomenon is a unique biological feature of *Babesia*, which is transmitted transovarially, unlike *Theileria* parasites (Chauvin et al., 2009). Based on the observation of *B. major* in the developing oocytes of *H. punctata* by electron microscopy, it was expected that *Babesia* would initially attach to the tunica propria of the oocyte by secreting substances that may have cement-like properties (Morzaria et al., 1977). The tunica propria is a connective tissue sheath comprised of four or five layers of finely fibrillary material, extending around each oocyte and covering it as the oocyte develops and enlarges (Ogihara and Taylor, 2014). The tunica propria is believed to be permeable to Vg and other hemolymph proteins that are taken up by the oocytes. VgR is localized in the outer surface membrane, likely on the tunica propria, of *H. longicornis* oocytes (Boldbaatar et al., 2008). Thus, the tunica propria is a key cell structure for Vg uptake for oocyte development as well as *Babesia* infection. It is also believed that oocytes are usually infected with *Babesia* early in their maturation process, probably before egg shell formation (Morzaria et al., 1977). Shell formation accelerates greatly during stage III and is completed by the end of stage IV of oocyte development (Ogihara and Taylor, 2014). Previously, we showed that stage I and II oocytes develop to mature stages at the preovipositional period and that active uptake of Vg *via* VgR is required for the development from stage III to stage IV during oogenesis (Mihara et al., 2018; Umemiya-Shirafuji et al., 2019). Maeda et al. (2016) reported that the *B. ovata* gene is present and is likely to increase 1 day after engorgement in the ovary, indicating that *Babesia* might first invade immature oocytes in the ovary at the early phase of the preovipositional period, such as 1–2 days after engorgement. Taken together, 1–2 days after engorgement is a suitable timing to assess the molecular interactions during the migration of *Babesia* from the midgut to other organs including the ovary in *H. longicornis*.

Three *Vg* genes have been identified from parthenogenetic *H. longicornis* (Boldbaatar et al., 2010c): HlVg-1 is synthesized in the midgut, HlVg-2 is synthesized in the fat body and ovary and HlVg-3 is synthesized in the fat body. In the present study, *HlVg-2* and *HlVg-3* mRNA expression in the fat body were not affected by *B. ovata* infection two to four days after engorgement (**Figure 1**). We then analyzed *HlATG6* and *HlVgR*, molecules involved in Vg uptake from the hemolymph into the ovary (Kawano et al., 2011), and found that their expression levels in *B. ovata*-infected ticks were lower than that in uninfected ticks 2 days after engorgement (**Figure 3**). It is believed that *HlATG6* play a role in endocytosis in Vg uptake from hemolymph to oocytes during oogenesis (Kawano et al., 2011). Subsequent western blot analyses revealed that the HlVg-2 protein accumulated in both the fat body and hemolymph of the *B. ovata*-iRBC group (**Figure 4**). These data of *HlVgR* and *HlATG6* gene expressions and HlVg-2 protein detection suggest that Vg uptake from the hemolymph to the ovary was suppressed in *B. ovata*-infected ticks 2 days after engorgement, compared with the control ticks. It was also found the discordance between *HlVgR* and *HlATG6* expression patterns after 3 and 4 days after engorgement. To better understanding of this phenomenon, localization of HlVgR and HlAtg6 proteins in the oocytes will be studied using *B. ovata*-infected *H. longicorni*s tissue sections in the future. Additionally, the accumulation of HlVg-2 in the hemolymph likely promoted HlVg-2 synthesis within the ovary because the Vg protein is required for oocyte maturation. Our previous study showed that there was no significant difference in the amount of eggs per body weight of engorged female ticks between *Babesia*-infected and uninfected ticks (Umemiya-Shirafuji et al., 2017). Thus, the promotion of HlVg-2 synthesis in the ovary of *B. ovata*-infected ticks likely contributed to the successful egg production to overcome reduced Vg uptake into the ovary. Although the individual function and relative contribution of each HlVg to oogenesis and/or *Babesia* infection are not yet clear, our current data emphasize that the roles of Vgs in *Babesia* transmission of vector ticks need to be further investigated.

Akt, a serine–threonine kinase, plays a central role in protein synthesis, glucose metabolism, and cell proliferation (Manning and Toker, 2017). Akt is also a downstream factor of the insulin signaling pathway that controls innate immunity in vertebrates and invertebrates (Luckhart and Riehle, 2007). In *H. longicornis*, mRNA expression of *Akt* was found in the fat body, ovary, midgut, and salivary glands (Umemiya-Shirafuji et al., 2012b). Knockdown of *Akt* mediated by RNAi led to the reduction of *HlVg* gene expression in the fat body, ovary, and midgut, suggesting that *Akt* is an upstream factor of Vg synthesis in ticks. In the present study, the expression level of the *Akt* in the *B. ovata*-iRBC group was higher than that in the RBC group 1–3 days after engorgement (**Figures 1**, **2**). It has been reported that the infection rate of *P. falciparum* was decreased in *A. stephensi* overexpressing *Akt* (Corby-Harris et al., 2010; Luckhart et al., 2013). The protozoan parasite *Trypanosoma cruzi* regulates their survival by increasing the expression of *Akt* in the host cells and by suppressing the expression of apoptosis-promoting factors, suggesting that such regulation is one of their survival strategies in the host body (Chuenkova and PereiraPerrin, 2009). Therefore, the increased *Akt* expression in *B. ovata*-iRBC ticks in our study might be attributed to a response against protozoan infection or a survival strategy of *Babesia* in the tick body. Moreover, *HlS6K* encoding S6K, a downstream factor of Akt, was upregulated in the fat body of *Babesia*-iRBC group 1 and 2 days after engorgement. In the ovary, upregulation of *HlAkt*, *HlTOR*, and *HlGATA* mRNAs was found in the *Babesia*-iRBC group 3 days after engorgement. These data indicate the promotion of protein synthesis in each organ; however, it is unclear whether the upregulation caused Vg synthesis specifically in *Babesia*-infected ticks. Analyses of activation of Akt signaling pathway molecules will be required to discuss their functions in *Babesia*-infected ticks.

**Figure 6:**
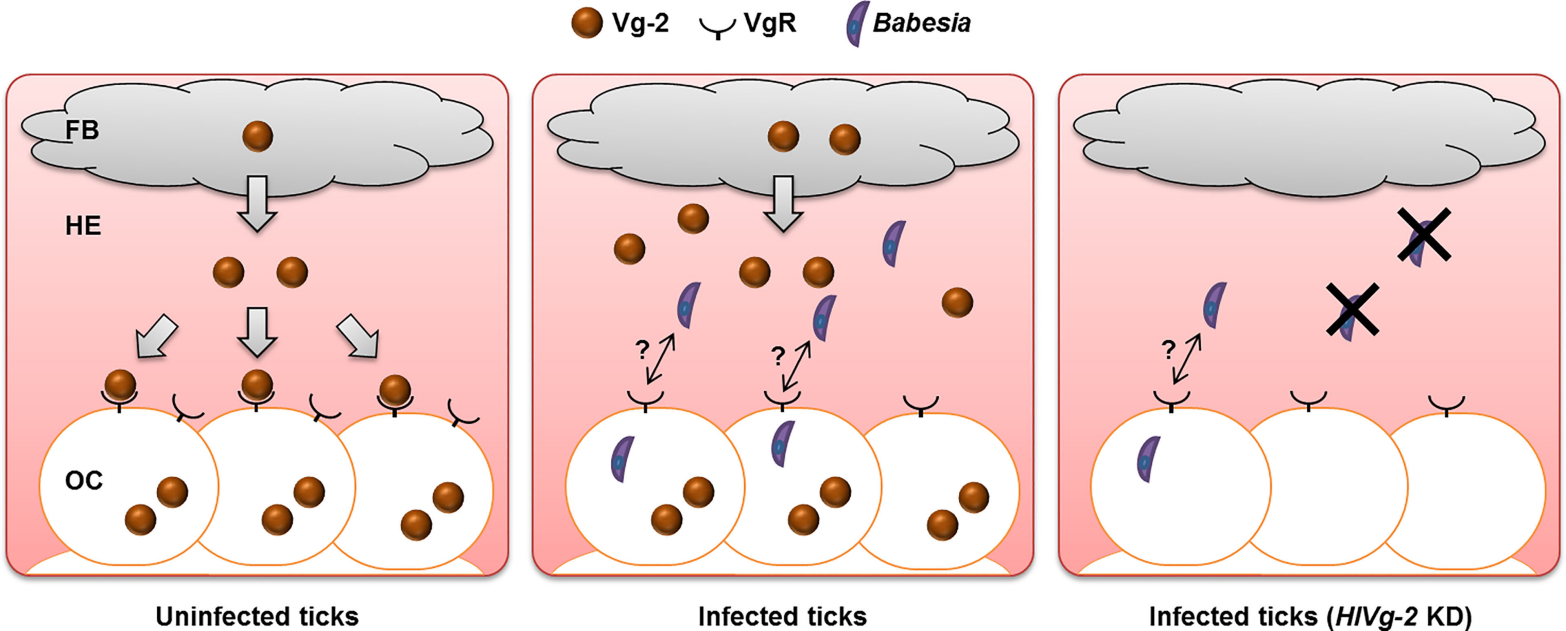
*Babesia* transmission model at the early phase of preovipositional period in parthenogenetic *H. longicornis*. In uninfected ticks, Vg-2 synthesized in the fat body is released into the hemolymph and is taken up by oocytes *via* VgR. In the presence of *Babesia*, Vg-2 accumulates in both the fat body and hemolymph. Although Vg-2 uptake *via* VgR from the hemolymph is suppressed, Vg-2 is synthesized within oocytes for their development. In *Vg-2* KD ticks, the amount of *Babesia* in the hemolymph decreased, but some *Babesia* invaded oocytes. FB, fat body; HE, hemolymph; KD, knockdown; OC, oocyte; Vg-2, vitellogenin-2; VgR, vitellogenin receptor.

Analyses at the gene and protein levels demonstrated that HlVg-2 uptake from the hemolymph into the ovary was suppressed in *B. ovata*-iRBC ticks 2 days after engorgement, compared with RBC ticks. These results raise the possibility that HlVg-2 might interact with *B. ovata* in the hemolymph of *H. longicornis* (**Figure 6**). Thus, we attempted to detect *B. ovata* in the hemolymph, ovary, and carcass of *HlVg-2*-knockdown ticks. Nested PCR revealed that the detection rate of *Babesia* in the ovary decreased in *HlVg-2*-knockdown ticks compared to control ticks. Furthermore, the relative DNA level of *B. ovata* in the hemolymph declined significantly in *HlVg-2* knockdown ticks. Previously, Boldbaatar et al. (2008) reported that in conventional PCR, *B. gibsoni* DNA was not detected in the eggs laid by *HlVgR* knockdown ticks after blood feeding on *B. gibsoni*-infected dogs. Therefore, *Babesia* survival in the hemolymph and/or *Babesia* invasion/infection in the oocytes might be affected by the presence of HlVg-2 and HlVgR. These strongly support our hypothesis that Vg and VgR are key molecules for transovarial transmission of *Babesia* in the vector tick. It is well-known that *Babesia* zygotes develop and proliferate in the midgut epithelial cells and produce kinetes into the hemolymph. The primary kinetes move to other tissues by floating in the hemolymph for the production of secondary kinetes (Jalovecka et al., 2018). Our data showing the decrease in relative DNA levels of *B. ovata* in the hemolymph (**Figure 5**) suggests that HlVg-2 is required for the presence or migration of *B. ovata* kinetes in ticks. Additionally, the decreased *B. ovata* DNA levels in the hemolymph might bring about decreased detection rates of *B. ovata* in the ovary of *HlVg-2* RNAi ticks. Furthermore, our results suggest that HlVg-2 interfered with *B. ovata* 2 days after engorgement. *Babesia* may pass through the midgut after schizogony within 24 h post engorgement and then migrate to the hemolymph (Maeda et al., 2016). Our results correspond to the time course of *Babesia* transmission in *H. longicornis* proposed by Maeda et al. (2016). Further studies are needed to elucidate the relationship between Vg, VgR, and *Babesia* in *H. longicornis*.

Three Vgs are essential for oogenesis (Boldbaatar et al., 2010b). We found that *Vg-2* expression was affected by *B. ovata* infection in *H. longicornis*, meaning that HlVg-2 is involved not only in oogenesis but also in the survival of *B. ovata* in the hemolymph and/or invasion into oocytes. Vg has been reported to act as an immune system in various arthropods. In honeybees, Vg binds to the membrane surface of bacteria and fungi and has bactericidal action (Park et al., 2018). It has been reported that Vg also binds to bacteria and possesses bactericidal and opsonic effects in fish (Li et al., 2008; Li et al., 2009). Mosquito Vg inhibits Thioester-containing protein 1, which is involved in the elimination of *Plasmodium*, so knocking down Vg has been shown to reduce the number of *Plasmodium* in mosquitoes (Rono et al., 2010). Recent studies have also shown that viruses and bacteria bind to arthropod Vg and invade the ovaries (Wei et al., 2017; Guo et al., 2018; Huo et al., 2018). It remains unclear whether the increased amount of HlVg-2 in the hemolymph possesses anti-*Babesia* activity or binding activity to *Babesia* to transport to other tissues (**Figure 6**). We also aimed to morphologically observe *B. ovata* in the *HlVg-2* RNAi tick samples; however, it was difficult to find the parasites in the present study. To find new strategies to combat *Babesia* spread by vector ticks, we will study the functions of oogenesis-related molecules, including other Vgs, in *Babesia*-infected ticks at the protein and cellular levels while also improving the experimental tools.

## Data Availability Statement

The sequence data used in this study can be freely and openly accessed at the DNA DataBank of Japan (DDBJ)/ European Nucleotide Archive (ENA)/GenBank: AB634844 for B. ovata ß-tubulin (https://www.ncbi.nlm.nih.gov/nuccore/AB634844.1/), AB601888 for HlAkt (https://www.ncbi.nlm.nih.gov/nuccore/AB601888), AB716688 for HlTOR (https://www.ncbi.nlm.nih.gov/nuccore/AB716688), AB491581 for HlS6K (https://www.ncbi.nlm.nih.gov/nuccore/AB491581), AB491580 for HlGATA (https://www.ncbi.nlm.nih.gov/nuccore/AB491580), AB359901 for HlVg-2 (https://www.ncbi.nlm.nih.gov/nuccore/AB359901), AB359902 for HlVg-3 (https://www.ncbi.nlm.nih.gov/nuccore/AB359902), AB299015 for HlVgR (https://www.ncbi.nlm.nih.gov/nuccore/AB299015), AB601889 for HlATG6 (https://www.ncbi.nlm.nih.gov/nuccore/AB601889), and EU048401 for HlP0 (https://www.ncbi.nlm.nih.gov/nuccore/EU048401).

## Ethics Statement

The animal study was reviewed and approved by The Obihiro University of Agriculture and Veterinary Medicine Animal Care and Use Committee.

## Author Contributions

MK and RU-S designed this study. MK, NS, and RU-S performed the experiments and the data analysis and drafted the manuscript. NY, S-IK, XX, HS, and KF participated in the study design and coordination and revised the manuscript. All authors actively contributed to the interpretation of the findings and read and approved the final manuscript.

## Funding

This work was supported by the Japan Society for the Promotion of Science (JSPS) KAKENHI Grant Numbers 16K18794, 18H02336, 19H03120, and 19K06416 and the Oshimo Foundation. This work was partially supported by the Ministry of Education, Culture, Sports, Science and Technology (MEXT) of Japan as a project for the Joint Usage/Research Center and Strategic International Collaborative Research Project (JPJ008837) Promoted by the Ministry of Agriculture, Forestry and Fisheries, Japan.

## Conflict of Interest

The authors declare that the research was conducted in the absence of any commercial or financial relationships that could be construed as a potential conflict of interest.

## Publisher’s Note

All claims expressed in this article are solely those of the authors and do not necessarily represent those of their affiliated organizations, or those of the publisher, the editors and the reviewers. Any product that may be evaluated in this article, or claim that may be made by its manufacturer, is not guaranteed or endorsed by the publisher.
